# Phenotyping of COPD with MRI in comparison to same-day CT in a multi-centre trial

**DOI:** 10.1007/s00330-024-10610-0

**Published:** 2024-02-12

**Authors:** Sebastian Nauck, Moritz Pohl, Bertram J. Jobst, Claudius Melzig, Hagen Meredig, Oliver Weinheimer, Simon Triphan, Oyunbileg von Stackelberg, Philip Konietzke, Hans-Ulrich Kauczor, Claus P. Heußel, Mark O. Wielpütz, Jürgen Biederer, on behalf of the COSYCONET Study Group

**Affiliations:** 1 Department of Diagnostic and Interventional Radiology, University Hospital of Heidelberg, Im Neuenheimer Feld 420, 69120 Heidelberg, Germany; 2 Translational Lung Research Center Heidelberg (TLRC), Member of the German Center for Lung Research (DZL), Im Neuenheimer Feld 130.3, 69120 Heidelberg, Germany; 3 Institute of Medical Biometry, University Hospital of Heidelberg, Im Neuenheimer Feld 130.3, 69120 Heidelberg, Germany; 4 Department of Neuroradiology, University Hospital of Heidelberg, Im Neuenheimer Feld 400, 69120 Heidelberg, Germany; 5 Department of Diagnostic and Interventional Radiology with Nuclear Medicine, Thoraxklinik at the University of Heidelberg, Röntgenstraße 1, 69126 Heidelberg, Germany; 6 Faculty of Medicine, University of Latvia, Raina bulvaris 19, Riga, LV-1586 Latvia; 7 Faculty of Medicine, Christian-Albrechts-Universität zu Kiel, 24098 Kiel, Germany

**Keywords:** Magnetic resonance imaging, Computed tomography, Chronic obstructive pulmonary disease, Pulmonary emphysema

## Abstract

**Objectives:**

A prospective, multi-centre study to evaluate concordance of morphologic lung MRI and CT in chronic obstructive pulmonary disease (COPD) phenotyping for airway disease and emphysema.

**Methods:**

A total of 601 participants with COPD from 15 sites underwent same-day morpho-functional chest MRI and paired inspiratory-expiratory CT. Two readers systematically scored bronchial wall thickening, bronchiectasis, centrilobular nodules, air trapping and lung parenchyma defects in each lung lobe and determined COPD phenotype. A third reader acted as adjudicator to establish consensus. Inter-modality and inter-reader agreement were assessed using Cohen’s kappa (im-κ and ir-κ).

**Results:**

The mean combined MRI score for bronchiectasis/bronchial wall thickening was 4.5/12 (CT scores, 2.2/12 for bronchiectasis and 6/12 for bronchial wall thickening; im-κ, 0.04–0.3). Expiratory right/left bronchial collapse was observed in 51 and 47/583 on MRI (62 and 57/599 on CT; im-κ, 0.49–0.52). Markers of small airways disease on MRI were 0.15/12 for centrilobular nodules (CT, 0.34/12), 0.94/12 for air trapping (CT, 0.9/12) and 7.6/12 for perfusion deficits (CT, 0.37/12 for mosaic attenuation; im-κ, 0.1–0.41). The mean lung defect score on MRI was 1.3/12 (CT emphysema score, 5.8/24; im-κ, 0.18–0.26). Airway-/emphysema/mixed COPD phenotypes were assigned in 370, 218 and 10 of 583 cases on MRI (347, 218 and 34 of 599 cases on CT; im-κ, 0.63). For all examined features, inter-reader agreement on MRI was lower than on CT.

**Conclusion:**

Concordance of MRI and CT for phenotyping of COPD in a multi-centre setting was substantial with variable inter-modality and inter-reader concordance for single diagnostic key features.

**Clinical relevance statement:**

MRI of lung morphology may well serve as a radiation-free imaging modality for COPD in scientific and clinical settings, given that its potential and limitations as shown here are carefully considered.

**Key Points:**

*• In a multi-centre setting, MRI and CT showed substantial concordance for phenotyping of COPD (airway-/emphysema-/mixed-type).*

*• Individual features of COPD demonstrated variable inter-modality concordance with features of pulmonary hypertension showing the highest and bronchiectasis showing the lowest concordance.*

*• For all single features of COPD, inter-reader agreement was lower on MRI than on CT.*

**Supplementary Information:**

The online version contains supplementary material available at 10.1007/s00330-024-10610-0.

## Introduction

Chronic obstructive pulmonary disease (COPD) is a common respiratory disorder, which globally caused 3.28 million deaths in 2019 [1]. For more than 50 years, COPD drug development aimed preferentially at improvement of spirometry indices as study end point [2]. However, since manifestation of the disease is inhomogeneous with different severity of damage to airways and lung parenchyma, global parameters from pulmonary function tests may remain normal or almost normal for a long time due to compensation of local functional impairment by intact portions of the organ [2, 3]. More recent concepts are focused on earlier treatment and take into account that different disease manifestations or phenotypes such as “emphysema-type” and “airway-type” show different rates of disease progression and require different treatment strategies [2, 4–8]. In the last two decades, computed tomography (CT) has become the method of choice for image-based phenotyping of COPD [7, 9]. Several multi-centre cohort studies employing CT for morphologic analysis of lung structure and phenotyping of COPD have improved the understanding of disease mechanisms in COPD and provided new biomarkers for the characterization of lung disease as well as clinical decision-making in the future [7, 9–12].

To avoid cumulative radiation exposure from CT for repeat monitoring, magnetic resonance imaging (MRI) has been suggested as a radiation-free non-invasive alternative. It combines imaging of morphological abnormalities with techniques for the assessment of regional lung function, as shown in monocentric COPD studies [13–15]. Moreover, in patients with cystic fibrosis, morpho-functional MRI and a suitable semi-quantitative scoring system for chronic-obstructive airway disease have already been established for clinical routine [15–21]. For imaging COPD, multi-centre experience with morpho-functional MRI regarding feasibility and agreement with CT was still missing. Thus, the purpose of the present study was to prospectively evaluate the feasibility and diagnostic yield of MRI for phenotyping of COPD and to compare it to CT in a large cohort study.

## Methods/design

### Study design

The trial (trial registration: German Clinical Trials Register DRKS00005072) was embedded into the German “Impact of Systemic Manifestations/Comorbidities on Clinical State, Prognosis, Utilisation of Health Care Resources in Patients with COPD” study (COSYCONET, NCT01245933), substudy: “Image-Based Structural and Functional Phenotyping of the COSYCONET Cohort Using MRI and CT” (MR-COPD, NCT02629432). COSYCONET is a prospective multi-centre study which has enrolled more than 2700 subjects [22, 23].

The present imaging sub-study was designed to examine the diagnostic value of lung MRI as a radiation-free alternative to low-dose CT (LDCT) for phenotyping of COPD. To examine concordance between MRI and CT, 607 participants from the COSYCONET cohort were enrolled at 15 COSYCONET study centres over a time span of 3 years (for the detailed inclusion criteria of the cohort study see [23]); additional exclusion criteria for the imaging sub-study are provided in the Supplementary Material.

This study was carried out in accordance with the rules of good clinical practice defined by the World Medical Association [24, 25]. It was approved by the Institutional Review Boards of all participating study centres and the German Federal Office for Radiation Protection. Written informed consent was obtained from all participants.

### Magnetic resonance imaging

MRI examinations were performed on clinical MR systems with 1.5 T (Magnetom Aera, Avanto, Espree and Symphony [Siemens Healthineers]) or 3.0 T (Magnetom Trio [Siemens Healthineers] and Ingenia [Philips]). A basic MRI protocol for the assessment of structural and functional lung alterations in COPD patients was composed as suggested in previous work and adapted to the specifications of each scanner, as far as necessary [15, 26]. The total image acquisition time was approximately 30 min. MRI included morphological non-contrast-enhanced and contrast-enhanced sequences in in- and expiration and a dynamic contrast-enhanced series to study lung perfusion. The protocol was designed to be applicable at all scanners in the different locations of the study. Therefore, it was based on commercially available sequences like 3D gradient echo (GE) and fast spin echo sequences for morphological imaging. More recent developments such as ultra-short echo-time sequences were not yet included. Dynamic perfusion imaging was performed using a T1-weighted keyhole pulse sequence (dynamic contrast enhancement (DCE)) and 2 ml gadolinium-based contrast agent (Gadobutrol, 1 mmol/ml, Bayer AG) followed by a saline chaser [27, 28]. After contrast agent administration, the 3D GE acquisitions were repeated in in- and expiration, with additional fat saturation preparation for the transversal images. Further protocol details are listed in the Supplementary Material.

### Computed tomography

CT examinations were performed on clinical CT scanners of different manufacturers with at least 40-row detector arrays. The standardized non-enhanced LDCT protocol employed inspiratory and end-expiratory spiral acquisitions of the entire lung in thin collimation, as described in the supplementary material.

### Image assessment

Scientific image processing was performed at the central coordinating centre in Heidelberg. MR and CT images were evaluated visually using a multimodal OsiriX (OsiriX 64-bit, Pixmeo SARL) post-processing workstation (iMac 27″, Apple Inc.) and two 21″ certified medical image displays (Eizo Nanao Corporation). Two radiologists with 3 years of experience in pulmonary imaging analyzed the images independently. Both examinations from each patient were read separately by each reader and blinded to the images and results obtained with the other modality. A time span of at least 2 weeks was kept in between the reads of MRI and CT to minimise recall bias. Finally, the records of both first readers were reviewed by a third reader with more than 20 years of experience in pulmonary MRI as adjudicator to establish a consensus.

The semi-quantitative visual analysis was based on a modified scoring system of the COPD Gene CT Workshop Group and established scoring systems for MRI in cystic fibrosis and COPD [11, 14, 20]. Multiple MRI and CT features of large and small airways disease were rated binary or using a 3-point-scale for each lobe as illustrated in Supplementary Table 4.

On CT, bronchiectasis and bronchial wall thickening were reported separately, while on MRI, a single sum score was given for bronchiectasis and/or bronchial wall thickening, since reporting these separately was expected to be difficult. Collapses of main and/or lobar bronchi and for small airways disease, centrilobular nodules and air trapping on expiratory scans were reported for both modalities. Adapted to the diagnostic scope of each modality, mosaic attenuation was recorded on CT, while lung perfusion deficits were recorded on MRI. On CT, the presence and extent of emphysema in each lobe was evaluated with a further extended 5-point scale (Supplementary Table 4). On MRI, lung parenchyma defects were recorded as indicators of lung emphysema. Paraseptal emphysema, bullae and signs of pulmonary hypertension were reported for both modalities. The leading type of emphysema (centrilobular or perilobular) was determined on CT. Following the semi-quantitative evaluations, each reader was requested to categorise the COPD into “airway-type”, “emphysema-type” or “mixed-type”.

### Statistical analyses

The statistical analyses were conducted with R v.4.2.0 (R Foundation for Statistical Computing). Mean ± standard deviation (SD), median and range as well as absolute and relative frequencies were used to describe the endpoints. Results from the adjudicator-established consensus served as the standard of reference to assess concordance between MRI and CT. Inter-modality and inter-reader variability were evaluated with Cohen’s Kappa (κ), which was rated as previously described [29]: κ < 0.00 = poor; 0.00 < κ ≤ 0.20 = slight; 0.21 < κ ≤ 0.40 = fair; 0.41 < κ ≤ 0.60 = moderate; 0.61 < κ ≤ 0.80 = substantial; 0.81 < κ ≤ 1.00 = near perfect. Additionally, the accuracy was calculated as the relative frequency of concordant evaluation. The reading results were managed using REDCap electronic data capture tools (Vanderbilt University) [30, 31].

## Results

**Fig. 1:**
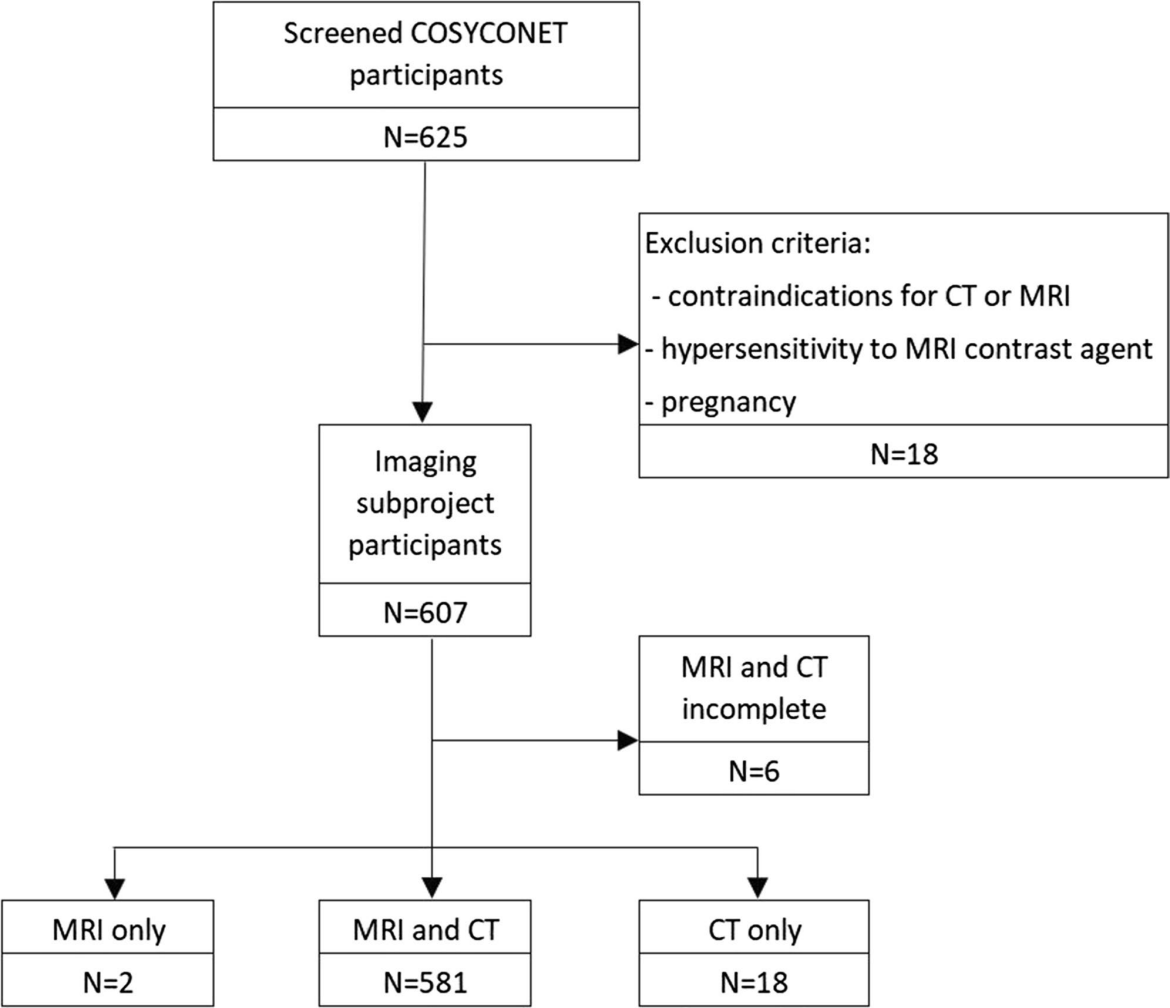
Study flow chart

A total of 607 participants were enrolled in the study, 581 (95.7%) of whom were examined with both MRI and CT while the remainder did not finish at least one of the examinations (e.g. because of claustrophobia) as illustrated in Fig. 1. The demographics and pulmonary function of the imaging cohort are shown in Table 1. The average radiation dose for in- and expiratory CT scans combined was 3.04 ± 0.68 mSv.

**Table 1:** Participant demographics, lung function and GOLD stages

	*n* (%)
Total participants	601
Male	368 (61.2)
Female	233 (38.8)
Age (years)	65.5 ± 8.6
Median	67
Range	42–85
BMI	26.8 ± 4.9
Smokers	552 (91.8)
Pack years	44.1 ± 35.9
FEV1%	59.6 ± 20.7
GOLD stages	
No COPD by GOLD criteria	84 (14)
GOLD stage 1	59 (9.3)
GOLD stage 2	249 (41.4)
GOLD stage 3	167 (27.8)
GOLD stage 4	39 (6.5)
No spirometry data	3 (0.5)

Participants were counted as smokers if they had smoked at least 100 cigarettes in their lifetime. *BMI* body mass index, *FEV1%* forced expiratory volume in 1 s percent predicted, *GOLD* Global Initiative for Chronic Obstructive Lung Disease

### Large airway disease

Mean sum scores for bronchiectasis and/or bronchial wall thickening on MRI and CT are presented in Table 2. The average per-lobe concordance of the combined bronchiectasis and/or bronchial wall thickening score on MRI with bronchiectasis on CT was slight (im-κ = 0.07), while the average per-lobe concordance with bronchial wall thickening on CT was fair (im-κ = 0.22, Table 3). On expiratory scans, collapses of each of the main bronchi were observed in 7–8% of participants on MRI and in 10% of participants on CT at a moderate inter-modality concordance. Collapses of lobar bronchi were observed in 6% of participants on MRI and in 19% of participants on CT at a fair inter-modality concordance (Table 4).

**Table 2:** Results of the sum scores for airway disease and emphysema on MRI and CT

MRI feature	Mean ± SD	Median (range)	ir-SD	ir-κ	CT feature	Mean ± SD	Median (range)	ir-SD	ir-κ	im-SD	im-κ
Bronchiectasis and/or bronchial wall thickening	4.5 ± 2.2	5 (0–10)	2.9	0.08	Bronchiectasis	2.2 ± 2.7	1 (0–12)	0.36	0.24	3.1	0.05
					Bronchial wall thickening	6 ± 2.2	6 (0–12)	0.28	0.13	2.2	0.11
Centrilobular nodules	0.15 ± 0.76	0 (0–10)	0.04	0.19	Centrilobular nodules	0.34 ± 1.2	0 (0–10)	0.83	0.32	1.1	0.26
Lung perfusion deficits	8.4 ± 2.7	9 (0–12)	0.44	0.27	Mosaic attenuation	0.37 ± 1.4	0 (0–12)	1.1	0.10		
Air trapping	0.40 ± 0.94	0 (0–6)	0.24	0.12	Air trapping	0.9 ± 1.5	0 (0–6)	0.77	0.24	1.3	0.31
Lung parenchyma defects	1.3 ± 2.1	0 (0–12)	0.44	0.27	Emphysema (3-p)	4 ± 3.5	5 (0–12)	2.5	0.39	2.9	0.13
					Emphysema (5-p)	5.8 ± 5.7	6 (0–22)	4	0.27		

Emphysema was evaluated on a 5-point-scale per lobe (5-p), which was reduced to a 3-point-scale (3-p) for comparison with MRI. *ir-SD* inter-reader standard deviation, *ir-κ* inter-reader concordance by Cohen’s κ, *im-SD* inter-modality standard deviation, *im-κ* inter-modality concordance by Cohen’s κ

**Table 3:** Cohen’s κ and accuracy of MRI and CT features on a lobar basis

CT feature	MRI feature	Cohen's κ							Accuracy					
		RLL	RML	RUL	LUL	Ling.	LLL		RLL	RML	RUL	LUL	Ling.	LLL
Bronchiectasis	Bronchiectasis/bronchial wall thickening	0.07	0.09	0.04	0.06	0.10	0.06		0.45	0.46	0.44	0.48	0.49	0.41
Bronchial wall thickening		0.30	0.14	0.24	0.25	0.17	0.31		0.75	0.65	0.65	0.64	0.60	0.77
Bronchial collapse	Bronchial collapse	0.29	0.11	0.07	0.33	0.10	0.23		0.90	0.95	0.96	0.99	0.97	0.86
Centrilobular nodules	Centrilobular nodules	0.41	0.25	0.22	0.21	0.09	0.39		0.95	0.95	0.95	0.96	0.96	0.94
Air trapping	Air trapping	0.40	0.32	0.36	0.37	0.38	0.37		0.78	0.94	0.94	0.95	0.95	0.76
Emphysema	Parenchymal defects	0.18	0.26	0.23	0.20	0.21	0.20		0.54	0.59	0.54	0.53	0.57	0.55

*RLL* right lower lobe, *RML* right middle lobe, *RUL* right upper lobe, *LUL* left upper lobe, *Ling*. lingula, *LLL* left lower lobe

**Table 4:** Results of binary airway and emphysema features that were assessed on MRI and CT

Feature	MRI (*n* = 583)		CT (*n* = 599)		Inter-modal	
	Prevalence (%)	ir-κ	Prevalence (%)	ir-κ	im-κ	Accuracy
Collapse right main bronchus	51 (9)	0.37	62 (10)	0.46	0.49	0.91
Collapse left main bronchus	47 (8)	0.40	57 (10)	0.46	0.52	0.92
Collapses of lobar bronchi	33 (6)	–0.02	115 (19)	0.16	0.27	0.84
Paraseptal emphysema	12 (2)	0.49	93 (16)	0.52	0.21	0.87
Bullae	26 (4)	0.60	70 (12)	0.55	0.48	0.92
Signs of pulmonary hypertension	124 (21)	0.65	125 (21)	0.63	0.84	0.95

On MRI and CT, a pulmonary trunc ectasia of 29 mm or more and a ratio of right ventricle diameter/left ventricle diameter of more than 1 were counted as signs of pulmonary hypertension. *ir-κ* inter-reader concordance by Cohen’s κ, *im-κ* inter-modality concordance by Cohen’s κ

### Small airways disease

**Fig. 2:**
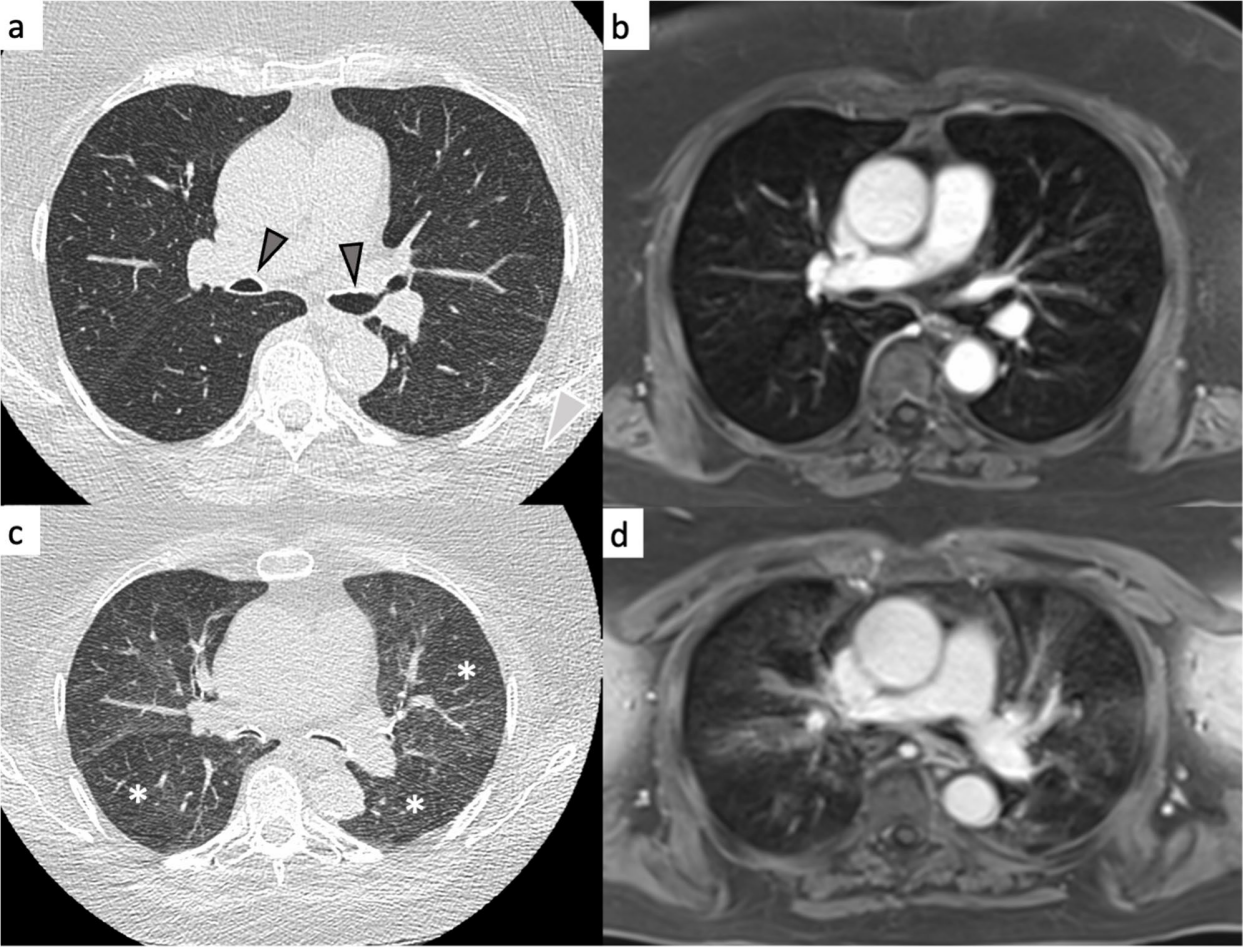
Large and small airway disease in a 70-year-old female participant (classified as airway predominant COPD phenotype). The CT images in in- and expiration (**a**, **c**) as well as the MRI in in- and expiration (**b**, **d**) show a collapse of the intermediate bronchus and left main bronchus with large areas of air trapping in expiration (asterisks)

On MRI, the mean sum scores for centrilobular nodules, lung perfusion deficits and air trapping were 0.15, 8.4 and 0.4, while on CT, the mean sum scores for centrilobular nodules, mosaic attenuation on inspiratory images and air trapping on expiratory scans were 0.34, 0.37 and 0.9, respectively (Table 2). The average per-lobe concordance between MRI and CT was fair for both centrilobular nodules (im-κ = 0.26) and air trapping (im-κ = 0.37, Table 3) (Fig. 2).

### Emphysema

On MRI, the mean sum score for lung parenchyma defects was 1.3/12, while on CT, the mean sum score for emphysema was 5.8/24. For comparison with MRI, the 5-point scale for the description of emphysema on CT was reduced to a 3-point scale as used for MRI. Based on this, per-lobe concordance for parenchymal defects on MRI with emphysema on CT showed a fair concordance (im-κ = 0.21, Table 3).

On MRI, paraseptal emphysema, bullae and signs of pulmonary hypertension were reported in 12, 26 and 124 of 583 subjects (CT 93, 70 and 125 of 599 subjects). Inter-modality concordance was fair for paraseptal emphysema (im-κ = 0.21), moderate for bullae (im-κ = 0.48) and near perfect for signs of pulmonary hypertension (im-κ = 0.84, Table 4). On CT, the leading type of emphysema was centrilobular in 375 of 599 subjects (63%), perilobular in 44 subjects (7%) and in 180 subjects (30%) the type of emphysema was not determined.

### Classification of COPD type

**Fig. 3:**
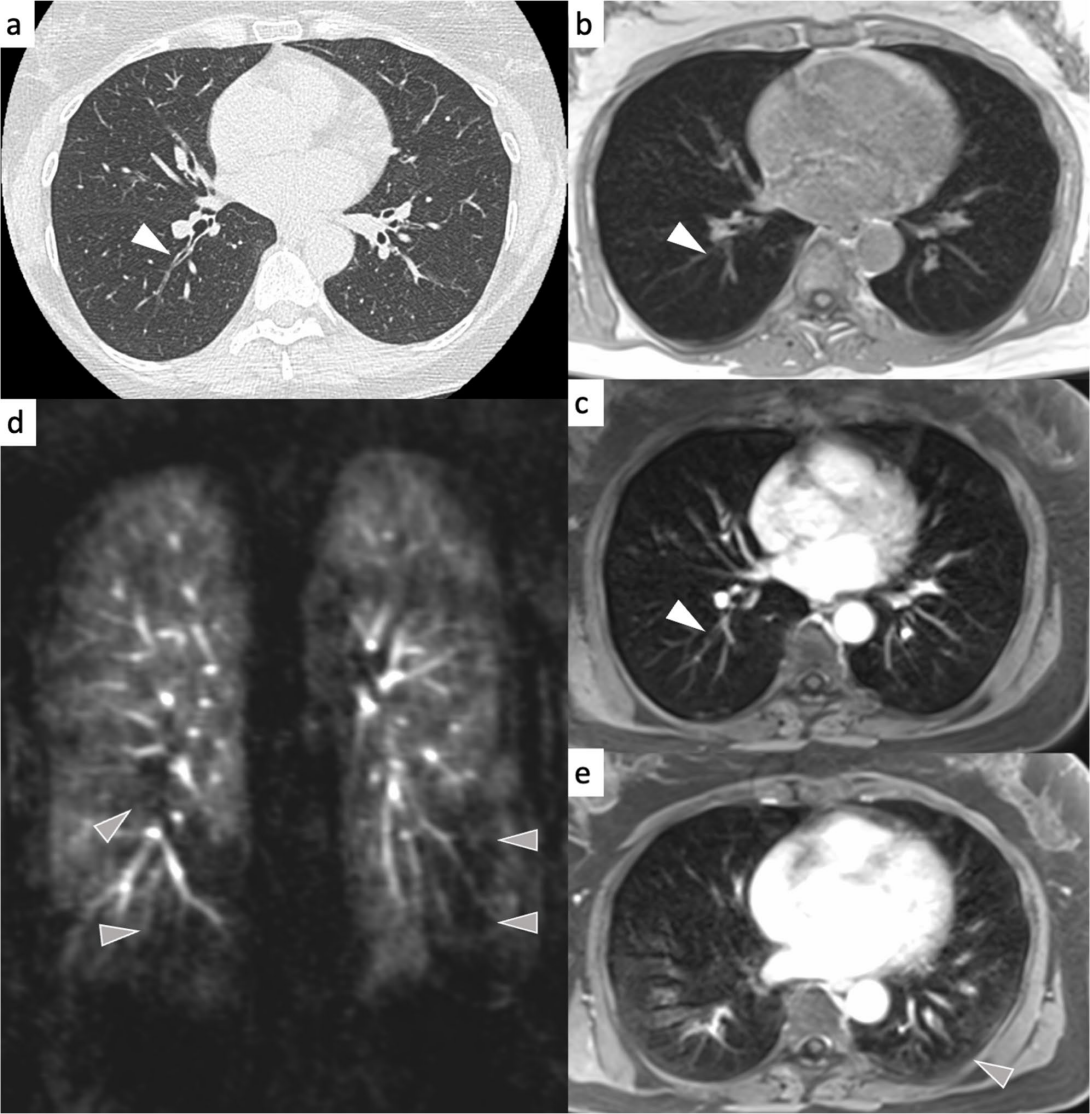
Airway-type COPD in a 57-year-old female participant (classified as airway predominant COPD phenotype). The white arrowhead on the CT image (**a**) indicates thickened bronchial walls in the right lower lobe. On the corresponding MRI, bronchial wall thickening appears less conspicuous with a slightly better visualisation on the contrast-enhanced transverse 3D gradient echo image (**c**) compared to the non-contrast-enhanced (**b**, white arrowheads). Functional deficits from airway disease appear as perfusion defects on the DCE series (**d**, grey arrowheads) and as air trapping on the contrast-enhanced transverse 3D gradient echo image in expiration (**e**)

**Fig. 4:**
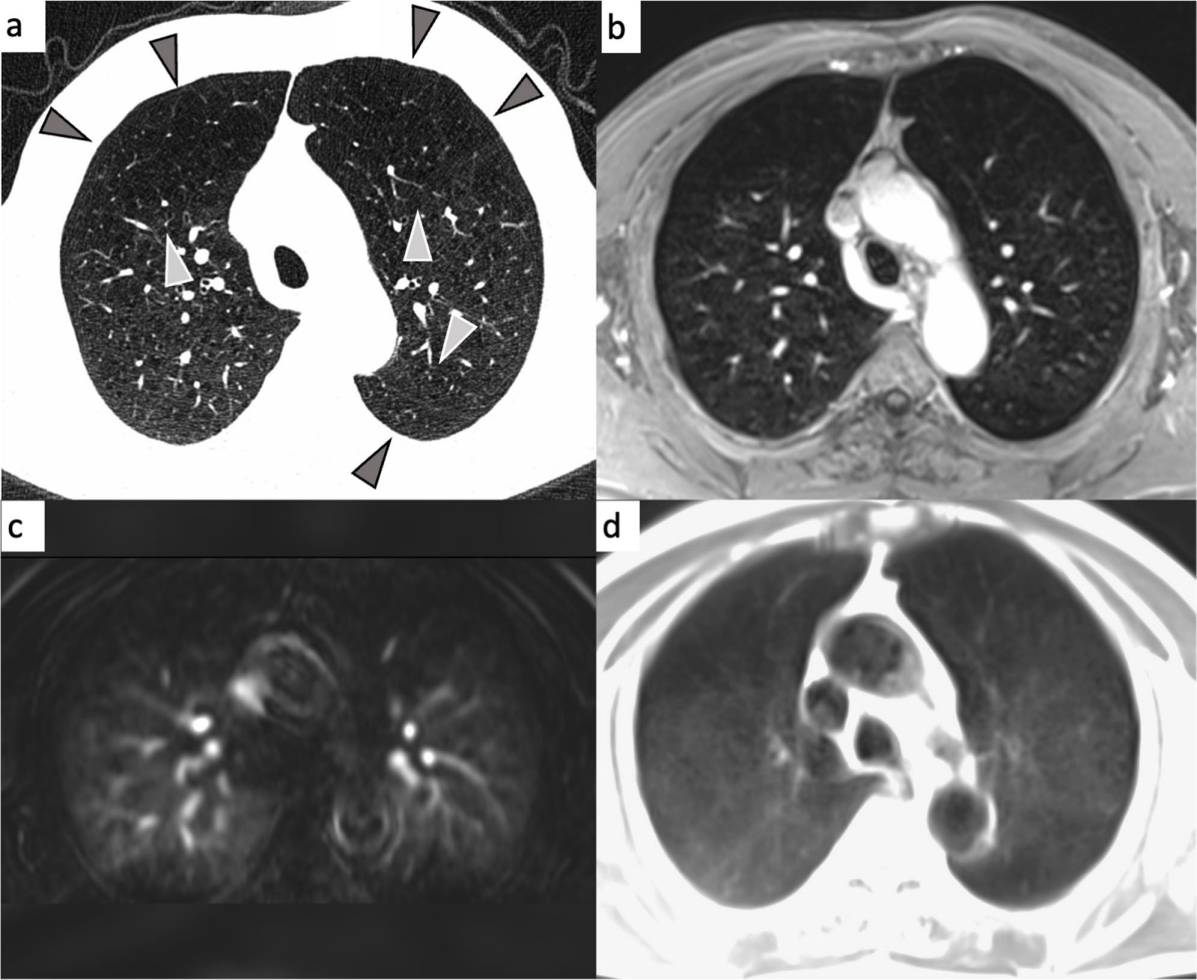
Emphysema-type COPD in a 69-year-old male participant (classified as emphysema predominant COPD phenotype). Arrowheads on the CT image (**a**) indicate severe emphysema in both anterior upper lung lobes and in the tip of the left lower lung lobe. On the corresponding MRI, the affected parts of the lung appear as lung parenchyma defects on the contrast-enhanced transverse 3D gradient echo image (**b**), as perfusion deficits in the DCE series (**c**) and as signal void in the transverse half Fourier fast spin echo image (**d**)

On MRI, “Airway-type”-COPD was observed in 370 of 583 subjects (63%), “Emphysema-type”-COPD was observed in 203 subjects (36%) and “mixed-type”-COPD was observed in 10 subjects (2%, ir-κ = 0.51). On CT, “Airway-type”-COPD was observed in 347 of 599 subjects (58%), “Emphysema-type”-COPD was observed in 218 cases (36%) and “mixed-type-COPD” in 34 cases (6%, ir-κ = 0.66). The inter-modality concordance regarding the COPD type was substantial at im-κ = 0.63 (Figs. 3, 4).

### Inter-reader agreement

**Fig. 5:**
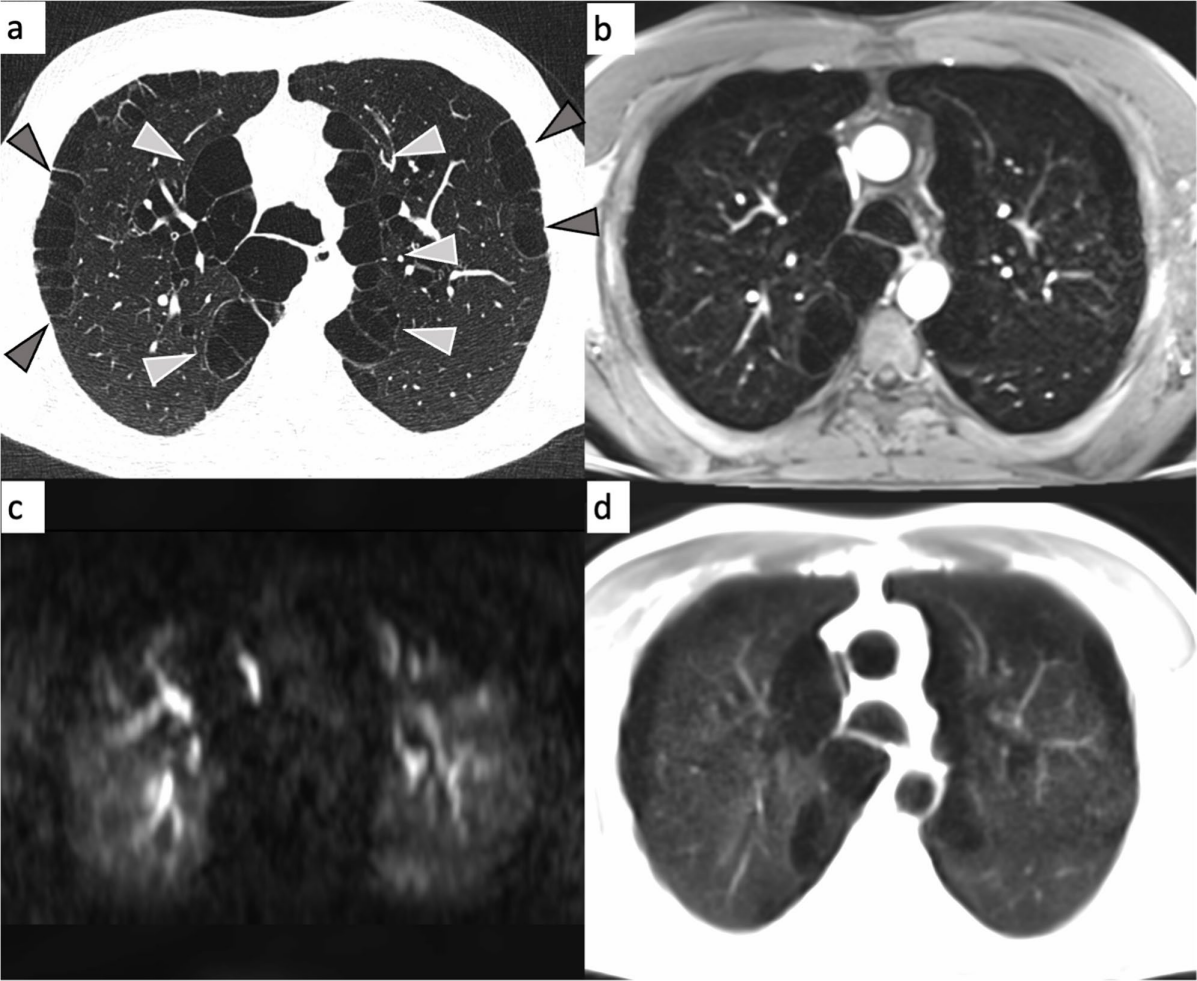
Paraseptal emphysema in a 51-year-old male participant (classified as airway predominant COPD phenotype). Arrowheads on the CT images (**a**) indicate areas of paraseptal emphysema with large bullae. On the corresponding MRI, the affected parts of the lung appear as lung parenchyma defects on the contrast-enhanced transverse 3D gradient echo image (**b**), as perfusion deficits in the DCE series (**c**) and as signal void in the transverse half Fourier fast spin echo images (**d**)

**Fig. 6:**
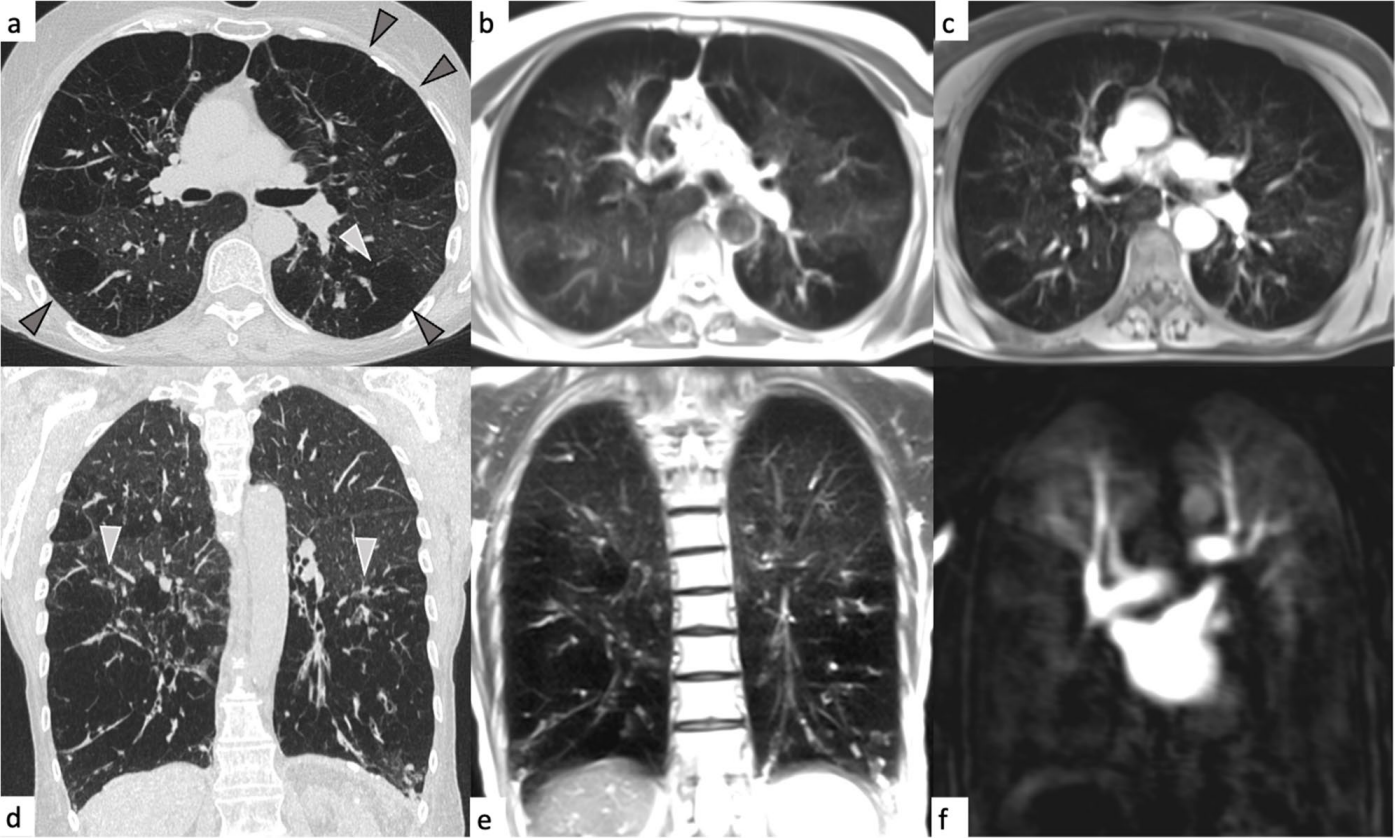
Mixed pattern with emphysema and extensive airway disease in a 62-year-old female participant (classified as airway predominant COPD phenotype). Arrowheads on the CT images (**a**, **d**) indicate severe emphysema in both anterior upper and posterior lung lobes. On the corresponding MRI, the affected parts of the lung appear as signal void in the transverse half Fourier fast spin echo images (**b**, **e**) or lung parenchyma defects on the contrast-enhanced transverse 3D gradient echo image (**c**) and as extensive perfusion deficits in the DCE series (**f**)

For all sum scores and binary features that were assessed, inter-reader concordance was lower on MRI compared with CT. For collapses of the main bronchi, collapses of lobar bronchi and air trapping, the inter-reader concordance of both MRI and CT was lower than that of the inter-modality concordance. For centrilobular nodules and COPD type, only the inter-reader concordance of MRI (but not of CT) was lower than the inter-modality concordance. “Collapses of lobar bronchi” was the only feature that showed no inter-reader concordance on MRI (ir-κ = −0.02); on CT, all features showed at least slight inter-reader concordance (Figs. 5, 6).

## Discussion

The conducted study shows the feasibility of lung MRI with a standardized imaging protocol in a multi-centre setting. Semi-quantitative visual evaluation of the images produced scores of key findings of COPD ranging from large over small airways disease to emphysema which allowed for a comparison of MRI and CT.

In large airways disease, inter-modality concordance was fair for bronchial wall thickening and slight for bronchiectasis on a lobar basis. This can mainly be attributed to the lower spatial resolution of MRI compared with CT. In the applied MRI protocols, the spatial resolution ranged from 1.04 × 1.04 to 1.76 × 1.76 mm in plane at a slice thicknesses of 1.8 to 6 mm. The spatial resolution of the CT scans was defined as 0.78 × 0.78 mm in plane with a slice thickness of 0.625 to 1 mm, thus producing 2 to 5 times better resolution in plane and approximately 5 to 40 times smaller voxel sizes. Of the key features of COPD, bronchiectasis of peripheral airways appears to be the most critical structure. Unless small airways appear with thickened walls or mucus filling, they can be well detected with CT, but no more with MRI [32, 33]. Since this was anticipated when setting up the study, only a combined sum score was planned for reporting bronchiectasis and/or bronchial wall thickening on MRI. Moreover, due to longer acquisition times, MRI is more sensitive to respiratory and cardiac motion, which further deteriorates the detection of fine structures [32]. It is therefore not surprising that bronchiectases were more frequently observed on CT. The difference in spatial resolution also explains that expiratory collapses of the main bronchi could be well seen on MRI, while inter-modality and inter-reader concordance were lower for lobar bronchi. Im-κ for collapses of the right and left main bronchi were 0.49 and 0.52, respectively, but on average only 0.27 on the lobar level. Similarly, inter-reader agreement dropped on CT from κ = 0.46 for collapses of the main bronchi to κ = 0.16 for collapses of lobar bronchi and on MRI from κ = 0.37–0.40 for collapses of the main bronchi to κ = 0.02 for collapses of lobar bronchi.

Regarding small airways disease, centrilobular nodules and air trapping mostly showed fair inter-modality concordance on a per-lobe basis, demonstrating that MRI can provide some information about small airways disease without visualizing small healthy airways themselves.

Emphysema only showed a slight to fair inter-modality concordance on a lobar level, which is most likely because lung tissue mainly consists of air-filled alveolar space with low hydrogen content and multiple air tissue surfaces, resulting in low signal intensity and poor signal-to-noise ratio [12, 34]. Therefore, an even lower signal intensity in emphysematous lung areas is difficult to perceive, which explains why so-called minus-pathology, such as emphysema, is difficult to detect with MRI.

Examination of the COPD type showed substantial inter-reader and inter-modality concordance, most likely because this was a binary feature that was assessed for the whole lung. Pulmonary trunk ectasia showed the highest inter-modality concordance (κ = 0.84), validating that large-vessel diameters can adequately be assessed by both MRI and CT.

On CT, inter-reader agreement ranged from κ = 0.10 for the sum score of mosaic attenuation to κ = 0.66 for classification of COPD type. Widell and Lidén reported kappa values of 0.28 to 0.85 for inter-reader agreement of pulmonary patterns on HRCT (high-resolution CT) with half of the patterns at kappa values above 0.70 [35]. That is significantly higher than the inter-reader agreements recorded in this study. Reasons for this could be the superior image quality of HRCT compared to LDCT and that the patterns of the HRCT study were on average easier to recognise. Moreover, the HRCT study recorded only binary findings while this study included scores with different options and semi-quantitative evaluation, hence potentially decreasing inter-reader agreement.

On MRI, almost all assessed hallmarks of COPD were underestimated with lower inter-reader agreement compared to CT. This can primarily be attributed to the lower spatial resolution and lower signal-to-noise ratio of MRI as explained above. Moreover, since lung MRI is still not a frequent examination in clinical routine, the readers, who had at least 3 years of experience in pulmonary imaging, were more experienced in reading CT compared to MRI, despite training sessions before the study reads. The relatively limited experience of the two primary readers compared with the adjudicator also most likely explains, why for bronchial collapses and air trapping, inter-reader concordance on CT and MRI each was lower than inter-modality concordance, which was based on the results from the consensus read.

In contrast to the COPDGene and EvA (emphysema vs airway disease) studies, which used two different CT scans for the analysis of the degree of emphysema and airway architecture (EvA) or the discrimination of emphysema and gas trapping (COPDGene), the present study used an identical LDCT protocol for inspiratory and expiratory scans in order to reduce radiation dose, obtain identical image quality and facilitate quality management procedures [36, 37].

One potential limitation of the study is the scoring system. For most features, it was based on a 3-point scale for absence or presence of a finding in less or more than 50% of a lung lobe. This produced rather a description of the presence of a finding than detailed quantitative information. For this study, this was considered appropriate and practicable, since COPD phenotyping is based on the presence or predominance of key features. Consequently, statistics focused on concordance of both methods for the presence of findings. Any calculated mean or median scores should therefore be interpreted carefully. The focus on qualitative rather than quantitative information also explains why concordance for attributing COPD phenotypes was higher than for most of the individual parameters.

Moreover, MR image quality in this study reflects a compromise and the best possible level that could be achieved with the installed MR scanners at the time of study setup in 2013 rather than the potential maximum with the latest equipment. Differences in MR scanners and overall image quality were obvious and documented with a dedicated MRI phantom [38]. Further analysis of the influence of scanner type, image quality and reader experience levels would be beyond the scope of this publication.

Finally, in this multi-centre study, image acquisition took place in 15 different locations, but the reading of all images was conducted in Heidelberg by the same 3 readers, which increased standardization. Having different readers at each location in the future might increase variance between the centres.

When discussing the benefits of avoiding radiation exposure by using MRI instead of CT, one might argue that most COPD patients are elderly with limited remaining life expectancy and that ionizing radiation from CT is therefore less relevant. However, this topic is disputable and controversely discussed, e.g. in the context of CT for lung cancer screening [39]. Nevertheless, COPD also affects younger patients and even if the percentage is small, it amounts to large absolute numbers given the high prevalence of COPD worldwide. For example, the prevalence of COPD in the USA in 2007–2012 in the age group “20–50 years” was about 1.6%, which is equivalent to over 2 million cases only in the USA [40]. Particularly for these patients, avoiding radiation exposure would be desirable.

Moreover, besides being radiation-free, MRI offers additional value compared to CT in terms of functional imaging. While the presented work focuses mainly on MRI of lung morphology, future applications will combine this approach with functional MRI of the lungs. It would be beyond the scope of this article to discuss all functional imaging capacities of MRI in detail, but approaches to study lung perfusion, airway dysfunction, lung ventilation and respiratory mechanics using periodic signal alterations from respiration and cardiac action, arterial spin labelling or aerosolized contrast agents, hyperpolarized or inert gases are ample [41]. It appears more than realistic that this will make lung MRI not only an alternative, but complementary to CT for imaging COPD in the future.

The functional imaging component of our study protocol used the clinically most established method which is first-pass contrast-enhanced imaging with bolus injection of gadolinium chelates and time-resolved gradient-echo sequences. Injection of gadolinium-based contrast materials is considered reasonably safe and no adverse reactions to contrast infection were recorded during the study. However, besides rare acute adverse reactions and even rarer potential long-term complications such as nephrogenic systemic fibrosis, repeat contrast-enhanced MRI scans may lead to gadolinium deposition in the brain [42]. No adverse events were observed and no clinical symptoms have been associated with gadolinium deposition in this study. The gadolinium retention can be minimized, but not completely prevented by using macrocyclic instead of linear agents [43]. Therefore, functional MRI techniques without intravenous contrast would be highly appreciated, but imaging results might be disappointing in COPD patients with emphysema and insufficient lung signal intensity [44].

Against the background of significant comorbidities and the high risk of lung cancer in COPD patients, it is well known that CT offers benefits such as opportunistic lung cancer screening or detection of coronary artery calcifications. For example, 91.8% of the participants of this study were current or former smokers. The sensitivity of MRI for the detection of lung nodules of at least 6 mm in diameter is over 70% and therefore only slightly lower compared to CT, with significantly inferior sensitivity of MRI for non-/subsolid nodules [45]. Thus, MRI offers moderate but still valuable opportunistic lung cancer screening capacities.

In conclusion, this study shows the potential and the limitations of lung MRI for COPD research in a multi-centre setting. Concordance of MRI and CT for phenotyping of COPD was substantial, while it was variable for individual key findings of COPD. Inter-modality concordance was fair to moderate for most features of large airway disease, but only slight to fair for bronchiectasis and emphysema while inter-reader agreement was generally lower on MRI compared to CT. This is mainly attributed to the lower spatial resolution of MRI, its limitations in detecting minus pathology and its susceptibility to motion artefacts. However, the information about regional manifestation of disease in the lungs from MRI could still be a valuable adjunct to clinical data and standard spirometry.

### Supplementary Information

Below is the link to the electronic supplementary material.

Supplementary file1 (PDF 211 KB)

## Abbreviations

BMI: Body mass index
COPD: Chronic obstructive pulmonary disease
CT: COMPUTED tomography
DCE: dynamic contrast enhancement
FEV_1_%: Forced expiratory volume in 1 s percent predicted
GE: Gradient echo
HRCT: High-resolution CT
im-κ: Inter-modality concordance by Cohens’s κ
ir-κ: Inter-reader concordance by Cohen’s κ
LDCT: Low-dose CT
MRI: Magnetic resonance imaging
SD: Standard deviation

## Declarations

### Guarantor

The scientific guarantor of this publication is Prof. Dr. Jürgen Biederer.

### Statistics and biometry

Dr. Moritz Pohl has significant statistical expertise.

### Study subjects or cohorts overlap

No study subjects or cohorts have previously been reported.

### Methodology

• prospective

• diagnostic study

• multicentre study
