# Supplementary material for: Phenotyping of COPD with MRI in comparison to same-day CT in a multi-centre trial
Source: Eur Radiol. 2024 Feb 12;34(9):5597–609. doi: 10.1007/s00330-024-10610-0 (PMC11364611; doi:10.1007/s00330-024-10610-0)
Supplement: Supplementary file 1 — Supplementary file1 (PDF 211 KB) [file 330_2024_10610_MOESM1_ESM.pdf]

Electronic supplementary material

Additional exclusion criteria compared to COSYCONET

For the imaging sub-study, the exclusion criteria were complemented excluding pregnant women and subjects with previous lung surgery (e.g. lung volume reduction or lung transplantation), subjects with moderate or severe exacerbations requiring antibiotic treatment within the last four weeks, acute psychosis or other conditions leaving the perception of the participant appear limited, and specific contraindications to MRI (MRI-incompatible implants such as pacemakers, lung volume reduction (LVR) coils, claustrophobia, acute and chronic renal insufficiency [GFR <40 ml / min according to Modification of Diet in Renal Disease (MDRD) calculation]).

Training of study centres and quality control

Study centres were preselected with the help of a standardized questionnaire, assessing the locally available technical pre-requisites for MRI and CT. For MRI, a field-strength of 1.5-3.0 T and gradient systems with a maximum strength of at least 33mT/m were considered to be eligible. Further, power-injectors for contrast application and capabilities for 4D perfusion measurement are required. For CT, multi-detector scanners with ≥40 detector rows were included with an equivalent dose of <3.5 mSv defined as a cut-off.

The MRI and CT protocols were adapted to the locally available scanners/hardware while maintaining comparable image quality. Regular phantom measurements were performed in each study centre. For MRI, in-house developed phantoms designed to mimic the signal properties of lung tissue, blood, muscle tissue and fat were manufactured and distributed to each centre[1]. The same MRI protocol used for the participant examinations was used for phantom measurements every three months. For CT, a single Catphan 600 (The Phantom

Laboratory) [2] was used for semi-annual CT measurements. Furthermore, standard operating procedures (SOPs) for performing MRI and CT scans, verbal instructions to subjects and pseudonymization of participant data were developed and if necessary, adapted to specific requirements of each study centre.

MRI acquisition

Most of the MRI sequences were to be acquired in inspiratory breath-holds, with several sequences additionally repeated in expiration. Images were acquired in coronal and transversal orientation. The examinations for morphological imaging of airways and parenchyma comprised fast 3D gradient-echo (GE) and fast spin echo (SE) sequences. The coronal GE sequence was repeated in an expiratory breath-hold. After this, a balanced steady state free precession GE sequence was acquired in free breathing followed by a T_2-weighted fast spin-echo series with periodically rotated overlapping parallel lines with enhanded reconstruction (PROPELLER) in multiple breath holds. An additional transversal ultra-fast SE sequence was acquired with a short T_1 inversion recovery (STIR) preparation.

Dynamic perfusion imaging was performed using a T_1-weighted keyhole pulse sequence (dynamic contrast enhancement, DCE) [3, 4] with a fixed dose of 2 ml gadolinium-based contrast agent (Gadobutrol, 1 mmol/ml, Bayer Vital GmbH) followed by a saline chaser. MR angiography was performed by subtraction of a native spoiled GE image acquired before the TRA measurement and a repeated measurement acquired after i.v. contrast bolus injection of Gadobutrol (0.1 mmol/kg body weight). After the administration of contrast agent, the fast 3D GE acquisitions were repeated in both ex- and inspiration, with additional fat saturation preparation for the transversal images. Breath-hold times were less than 20 seconds each. Detailed information on the applied sequence parameters is listed in

Supplementary Table 1 based on the protocol designed for 1.5T Siemens MAGNETOM Aera (Siemens Healthineers). All sequences are approved for clinical use and commercially available.

CT protocol

The CT protocol was defined according to the recommendations of the German Radiological Society (DRG) [3] employing a thin slice collimation of 0.6 mm, a pitch of 0.6-1.0, a tube potential of 120 kVp and a tube current of 35 effective mAs for most scanner types. Minor modifications were required to adjust the protocol to the specifics of a broad spectrum of scanner types from different manufacturers used in this study. A detailed CT acquisition protocol is presented in Supplementary Table 2. The low-dose CT protocol provided morphological information of clinical significance on pulmonary and extra-pulmonary structures, comparable to CT examinations in the context of lung cancer screening. The additional end-expiratory images allowed for improved differentiation between air-trapping caused by collapse or obstruction of small airways and emphysema [4]. In addition, valuable information on stability of the tracheobronchial system could be obtained. The maximum effective radiation dose of inspiratory and end-expiratory CT scans together was less than 3.5 mSv, which was comparable to CT examinations at that time in the context of lung cancer screening and significantly below the typical values for a standard chest CT with about 6 mSv. CT image reconstructions comprised three-dimensional datasets with thin slice thickness and smooth as well as edge-enhancing algorithms (Supplementary Table 3). All protocols were supported by commercially available CT devices.

Supplemental references

1. Triphan S, Biederer J, Burmester K, et al (2018) Design and application of an MR reference phantom for multicentre lung imaging trials. PLOS ONE 13:e0199148. https://doi.org/10.1371/journal.pone.0199148

2. Gulliksrud K, Stokke C, Martinsen ACT (2014) How to measure CT image quality: variations in CT-numbers, uniformity and low contrast resolution for a CT quality assurance phantom. Phys Med 30:521–526. https://doi.org/10.1016/j.ejmp.2014.01.006

3. Biederer J, Wildberger J, Reuter M, et al (2008) Protokollempfehlungen für die Computertomografie der Lunge. Rofo-fortschritte Auf Dem Gebiet Der Rontgenstrahlen Und Der Bildgebenden Verfahren - ROFO-FORTSCHR RONTGENSTRAHL 180:471–479. https://doi.org/10.1055/s-2008-1081445

4. Jörres RA, Welte T, Bals R, et al (2010) [Systemic manifestations and comorbidities in patients with chronic obstructive pulmonary disease (COPD) and their effect on clinical state and course of the disease--an overview of the cohort study COSYCONET]. Dtsch Med Wochenschr 135:446–449. https://doi.org/10.1055/s-0030-1249185

Supplementary Table 1. Base MRI protocol, designed for 1.5T Siemens MAGNETOM Aera.

Sequences	mode	orien-tation	breath mode	contrast agent	TR (ms)	TE (ms)	FoV (mm^2)	slice th. (mm)	voxel size (mm^2)	matrix	PA factor	scan time (min:s)
VIBE	3D	cor	insp	native	3.61	1.63	400×400	4.0	1.39×1.39	288×288	2	0:16
VIBE	3D	tra	insp		3.29	1.61	400×300	4.0	1.25×1.25	320×240	2	0:16
HASTE	2D	cor	insp		314.0	20.0	400×400	6.0	0.78×0.78	512×512	3	0:13
HASTE	2D	tra	2×insp		500.0	27.0	450×366	8.0	1.41×1.41	320×260	2	0:35
HASTE	2D	cor	exp		314.0	20.0	400×400	6.0	0.78×0.78	512×512	3	0:13
TrueFISP	2D	cor	free		448.9	1.17	400×400	4.5*	0.78×0.78	512×512	3	2:20
BLADE	2D	cor	5×insp		905.0	73.0	400×400	6.0	1.25×1.25	320×320	2	2:13
HASTE IRM	2D	tra	2×insp		502.0	72.0	400×400	6.0	1.56×1.56	256×256	2	0:38
FLASH	3D	cor	insp		2.80	1.04	350×400	1.8	1.04×1.04	336×384	3	0:16
TWIST	3D	cor	Insp	dynamic	1.73	0.76	366×450	5.0	1.76×1.76	208×256	2	0:37
FLASH	3D	cor	insp	angio	2.80	1.04	350×400	1.8	1.04×1.04	336×384	3	0:16
VIBE FatSat	3D	tra	Insp	post-contrast	3.29	1.61	400×300	4.0	1.25×1.25	320×240	2	0:17
VIBE FatSat	3D	tra	exp		3.29	1.61	400×300	4.0	1.25×1.25	320×240	2	0:17
VIBE	3D	cor	Insp		3.61	1.63	400×400	4.0	1.39×1.39	288×288	2	0:16
VIBE	3D	cor	exp		3.61	1.63	400×400	4.0	1.39×1.39	288×288	2	0.16

The parameters shown are acquisition **mode** (3D or 2D multi-slice), slice/slab **orientation**, **breath**ing **mode** (**insp**iration, **exp**iration or **free** breathing and number of breath-holds), status of **contrast agent** during acquisition, repetition time **TR**, echo time **TE**, **F**ield **o**f **V**iew, **slice th**ickness, in-plane resolution/**voxel size**, **matrix** size, **pa**rallelization **factor** and total **scan time**. * The slices in the balanced SSFP (TrueFISP) sequence were acquired with 60% overlap.

Supplementary Table 2. Acquisition parameters for paired inspiratory and expiratory CT.

Scanner models	Siemens Definition Siemens Definition AS 40/ 64/ Flash 128 GE Lightspeed VCT 64/ GE Optima 64/ Philips Brilliance 64/ iCT 256
Scan Type	Spiral
Rotation Time (s)	0.33 – 0.50 s
Collimation	40 / 64 / 128 x 0.6-0.625 mm
Pitch	0.6-1.0
kVp	120 kVp
mA	30 - 35 eff. mAs
Dose modulation	Off
Matrix	512 x 512
Calibration phantom	Air / water phantom / CatPhan
Max. eff dose/scan	< 1.75 mSv
Max. eff. overall dose	< 3.50 mSv

Supplementary Table 3. Image reconstruction protocol for paired inspiratory and expiratory CT acquisitions.

**Acquisition**	**Orientation**	**FOV**	**Slice thickness (mm)**	**Interval (mm)**	**Convolution kernel***
**Inspiratory**	axial	lung	1.25-1.50	0.70-0.75	B70f/LUNG/L
**Inspiratory**	axial	lung	0.625-1.00	0.50	B30f/SOFT/B
**Inspiratory**	axial	including soft tissue of torso	0.625-1.00	0.50	B30f/SOFT/B
**Expiratory**	axial	lung	0.625-1.00	0.50	B30f/SOFT/B

*Vendor-specific generic names for Siemens/GE/Philips

Supplementary Table 4. Standardized semi-quantitative visual evaluation of structural and functional lung alterations on MRI and CT images.

	MRI feature	MRI score	CT feature	CT score
**Large airway disease**	Bronchiectasis and/or bronchial wall thickening	0-2 points per lobe (0 = absent, 1 = ≤ 50%, 2 = >50% of the lobe affected), 0-12 points for the whole lung	Bronchiectasis	0-2 points per lobe (0 = absent, 1 = ≤ 50%, 2 = >50% of the lobe affected), 0-12 points for the whole lung
			Bronchial wall thickening	
	Collapse right main bronchus	binary, yes/no	Collapse right main bronchus	binary, yes/no
	Collapse left main bronchus		Collapse left main bronchus	
	Collapses of lobar bronchi		Collapses of lobar bronchi	
**Small airway disease**	Centrilobular nodules	0-2 points per lobe (0 = absent, 1 = ≤ 50%, 2 = >50% of the lobe affected), 0-12 points for the whole lung	Centrilobular nodules	0-2 points per lobe (0 = absent, 1 = ≤ 50%, 2 = >50% of the lobe affected), 0-12 points for the whole lung
	Lung perfusion deficits		Mosaic attenuation	
	Air trapping	binary for each lobe (0 = absent, 1 = present), 0-6 points for the whole lung	Air trapping	binary for each lobe (0 = absent, 1 = present), 0-6 points for the whole lung
**Emphysema**	Lung parenchyma defects	0-2 points per lobe (0 = absent, 1 = ≤ 50%, 2 = >50% of the lobe affected), 0-12 points for the whole lung	Emphysema	0-4 points per lobe (0 = <5%, 1 = 5-25%, 2 = 26-50%, 3 = 51-75%, 4 = >75%), 0-24 points for the whole lung
	Leading type of emphysema	Binary, centrilobular or panlobular	Leading type of emphysema	Binary, centrilobular or panlobular
	Paraseptal emphysema	Binary, yes/no	Paraseptal emphysema	Binary, yes/no
	Bullae		Bullae	
	Signs of pulmonary hypertension*		Signs of pulmonary hypertension*	

* On MRI and CT, a pulmonary trunc ectasia of 29 mm or more and a positive ratio of right ventricle diameter / left ventricle diameter were counted as signs of pulmonary hypertension.
